# The Dental Association Meetings

**Published:** 1883-09

**Authors:** 


### Editorial, Etc.

---

THE DENTAL ASSOCIATION MEETINGS.—During the past month the principal Associations have held their annual meetings—the Southern Dental Association, in Atlanta, Georgia, and the American Dental Association a week later at Niagara Falls The American Dental Society of Europe, also held its annual meeting at Cologne, which lasted for three days and has become quite an important annual event with the members of the profession sojourning in foreign countries.

The contributions of Dr. Miller, of Berlin, were very interesting and instructive, and added much to the importance of this European meeting.

The meeting of the Southern Association at Atlanta, was well attended and much interest manifested in the proceedings. It was very ably presided over by Dr. L. D. Carpenter, and his decisions met with universal approval. The Georgia State Society met at the same time and place, and the two meetings proved to be both instructive and important in their effects upon the advancement of professional interests in the Southern section of our country. The meeting of the American Association, at Niagara Falls, was a very successful one, and was largely attended. We notice the names of an unusual number, at least for recent years, of Southern representatives to this meeting, which we are pleased to note, and trust that our professional brethern from all sections will manifest an interest in all the different Association meetings of the land, and show by their presence that the object of such annual gatherings is being appreciated, and a spirit evinced that shall tend to materially benefit the members of the profession wherever located. The complaint, however, is general in regard to all these meetings for the present year, that while some of the papers read were of great merit, and showed careful study and research, the majority were not up to the standard, requisite for such intelligent bodies. With the hope that the future may correct this defect, we urge upon the able and intelligent members of the profession to come to the rescue for the honor of their calling.

